# Factors Affecting Psychiatric Bed Utilisation by People With Intellectual Disabilities: A Time Series Analysis Using the English National Mental Health Services Data Set

**DOI:** 10.1111/jir.70003

**Published:** 2025-06-23

**Authors:** Atiyya Nisar, Paul A. Thompson, Harm Boer, Haider Al‐Delfi, Peter E. Langdon

**Affiliations:** ^1^ Intellectual Disabilities Research Institute (IDRIS) University of Birmingham Birmingham UK; ^2^ Coventry and Warwickshire Partnership NHS Trust Coventry UK; ^3^ REACH OUT Provider Collaborative Adult Secure Care West Midlands UK; ^4^ Birmingham Community Healthcare NHS Foundation Trust Birmingham UK; ^5^ Herefordshire and Worcestershire Health and Care NHS Trust Worcester UK

**Keywords:** hospital admissions, intellectual disabilities, psychiatric beds, time series, transforming care

## Abstract

**Background:**

In 2015, the Building the Right Support programme was launched for England in an attempt to reduce the number of psychiatric inpatients with intellectual disabilities and/or autism by 35%–50%. This target, and subsequent targets, were missed, and for 2025–2026, the government further committed to reducing numbers by 10%. Considering these continued targets, we aimed to investigate psychiatric bed utilisation over time, and to further understand factors that may influence psychiatric admissions and discharges of people with intellectual disabilities and/or autism, by utilising time series modelling with national English data to explore the relationship between a set of chosen sociodemographic, clinical and service‐related predictor variables and the following outcome variables: (1) total monthly number of hospital spells, (2) total monthly number of discharges, (3) total monthly number of admissions, (4) ratio of community to non‐community discharges, (5) number of inpatients with a length of stay under 2 years, (6) number of patients with a length of stay over 2 years and (7) total number of distinct individuals who had been subjected to restraints.

**Methods:**

Using data from the publicly available Mental Health Services Data set, we utilised linear regression (with moving average or auto‐regressive errors) to examine the relationships between variables over time, from February 2013 to January 2024.

**Results:**

Over time, the number of inpatients decreased by an average of 4.55 patients per month. The number of inpatients with a length of stay greater than 2 years reduced over time. Periods of time when the number of inpatients was greater were associated with more inpatients under the age of 18 years. Periods of time when hospital stays, admissions and discharges were higher were associated with fewer White inpatients relative to non‐White inpatients. Periods of time with more patients detained under Part II of the Mental Health Act were associated with more admissions and the increased use of restraint.

**Conclusions:**

Over the last 11 years, the planned closure of psychiatric inpatient beds has been unsuccessful. Our findings indicated that periods of increased psychiatric bed utilisation were associated with more admissions of younger people, non‐White inpatients and those likely to be experiencing a crisis. Future research should explore how psychiatric beds can be utilised more effectively alongside community‐based services and long‐term trajectories using participant level data.

## 1 Background

NHS England (2015) introduced the Transforming Care initiative in response to the abuse and mistreatment of individuals with intellectual disabilities and/or autism within inpatient settings. One of the key goals of Transforming Care was to reduce the number of psychiatric inpatients by 35%–50% while increasing community‐based provision. These targets were missed, and a new target was set in 2019 within the NHS long‐term plan to reduce the number of inpatients by less than half of the number within hospital within the year 2015 (Department of Health and Social Care 2019). This target was also missed, and the government have now committed to reduce the number of inpatients by 10% within the years 2025–2026 (NHS England 2025). One of the reasons that these targets may have been continuously missed is likely due to the lack of effective investment within community‐based services, especially during an age of continuing austerity, which led to an increase in mental health problems across the country (Barr et al. 2015), and a reduction in community‐based services for people with intellectual disabilities (Forrester‐Jones et al. 2021). As a consequence, it is likely that more people with intellectual disabilities and/or autism have been at risk of mental health crisis necessitating admission to a psychiatric hospital within England since 2015.

Historically, across the world, deinstitutionalisation programmes have led to the closure of long‐stay institutions for people with intellectual disabilities promoting community inclusion (Holt et al. 2000). However, across the nations of the United Kingdom, there continues to be specialist state funded community health and inpatient psychiatric services for people with intellectual disabilities (Perera and Courtenay 2018), which is not the case within other countries, where there is a lack of such specialist provision (Holt et al. 2000; Kwok and Chui 2008; Lunsky et al. 2007; Melvin et al. 2022). There is some evidence to indicate that admission to specialist inpatient psychiatric provision for people with intellectual disabilities and/or autism is associated with superior outcomes compared to admission to generic inpatient mental health services (Melvin et al. 2022). Generally, there is evidence that psychiatric hospital admissions are associated with improvements in mental health among those with intellectual disabilities and/or autism (Melvin et al. 2022), and concerns have been raised as to whether reducing psychiatric bed utilisation is the most appropriate recourse (Hossain et al. 2020; Sheehan et al. 2015), particularly if there is insufficient resource and infrastructure in the community to meet demands. Arguably, it may be of greater benefit to focus upon the effective and appropriate use of psychiatric beds while attempting to provide a level of health and social community care that would preclude the need for psychiatric hospital admission for the majority. At the same time, it will be the case that individuals within society may experience mental health crisis necessitating detention under the Mental Health Act, 1983, within England and Wales; it is the case that everyone subject to detention are entitled to a psychiatric bed.

Within England and Wales, people can be removed from their community and placed in a psychiatric hospital if the nature and degree of their mental illness and associated risk warrants admission to a psychiatric hospital. This can be authorised under Part II of the Mental Health Act, 1983, by two medical doctors, one of whom is authorised under s.12 of the Act, and an additional Approved Mental Health Practitioner. These patients would likely to admitted to an acute mental health ward or an assessment and treatment unit, which is a specialist inpatient unit for only people with intellectual disabilities. Admission can also be authorised under Part III of the Act when someone appears before a judge due to having committed an indictable criminal offence who also has a mental illness, an intellectual disability, or autism. Admission under Part III of the Act often serves as a method to divert an individual with mental illness, autism, or intellectual disability away from prison and into psychiatric hospital care, and judges have the option to place such a patient under a Restriction Order. Restricted patients can only be discharged from hospital by a Mental Health Tribunal and the Secretary of State for Justice, rather than clinicians. These patients are most likely to be admitted to a special forensic unit, including specialist forensic units for only individuals with intellectual disabilities and/or autism.

Data about psychiatric bed utilisation in England are publicly available, including data about children and adults with intellectual disabilities or autism. Data are published monthly by NHS Digital to either (1) the Assuring Transformation (AT) dataset, which contains data about the utilisation of psychiatric beds commissioned specifically for people with intellectual disabilities and/or autism, or (2) the Mental Health Services Data Set (MHSDS), which is a national dataset of patients in contact with mental health services across England, includes data regarding monthly inpatient bed utilisation. A previous time series analysis undertaken by Langdon et al. (2023) made use of the AT dataset. They found that the number of inpatients with intellectual disabilities and/or autism in a psychiatric bed specifically commissioned for people with intellectual disabilities and/or autism in England had reduced over time by 21 or 24% from December 2013 to March 2021. This was dependent upon which group of data were used for the calculation, indicating that the target set by Transforming Care was missed. The authors also reported that an increase in the number of consultant psychiatrists over time was found to be associated with reductions in numbers of inpatients, also over time, while increases in pre‐admission Care (Education) and Treatment Reviews (C(E)TRs) were associated with increased admissions, and post‐admission C(E)TRs were associated with increased discharges over time. Taylor (2021) also reported that the Transforming Care bed closure targets were missed using data from the AT dataset. Taylor et al. (2017) also argued that closing psychiatric beds for people with intellectual disabilities and/or autism may lead to more of this group being admitted to prison. There is evidence that psychiatric bed closure is associated with an increase in the number of prisoners being transferred into psychiatric hospitals (Keown et al. 2019), and an overall increase in the prison population (Wild et al. 2022).

Considering the continuing aim to reduce the number of psychiatric inpatients with autism and/or intellectual disabilities within England, we completed a time series modelling study using data from the MHSDS from February 2018 to January 2023. Therefore, the aim of the current study was to examine psychiatric bed utilisation over time and to understand what sociodemographic, clinical and service‐related predictor variables may influence admissions and discharges of people with intellectual disabilities and/or autism to any type of psychiatric bed (e.g., forensic, acute mental health, psychiatric intensive care unit, assessment and treatment unit) across England, not just those commissioned solely for use by people with intellectual disabilities and/or autism. We utilised time series modelling to explore the relationship between a number of chosen sociodemographic, clinical and service‐related predictor variables taken from the MHSDS, and the following outcome variables, also taken from the MHSDS dataset: (1) total monthly number of hospital spells, (2) total monthly number of discharges, (3) total monthly number of admissions, (4) ratio of community to non‐community discharges, (5) number of inpatients with a length of stay under 2 years, (6) number of patients with a length of stay over 2 years and (7) total number of distinct individuals who had been subjected to restraints.

## 2 Methods

### 2.1 Data Extraction

The online MHSDS pertaining to inpatient psychiatric bed utilisation by people with intellectual disabilities and/or autism from February 2018 to January 2024 were downloaded from NHS Digital (2024). Data are aggregated by month and represent patient counts. Data are submitted to NHS Digital by NHS Trusts providing mental health services, acute care, specialist services for people with intellectual disabilities as well as independent sector health care providers, the voluntary sector, and any other provider of mental health care within England. Individual patient level data is submitted and aggregated data are placed within the public domain. These data are used for research and audit purposes, inspection, regulation, and the commissioning of services. The MHSDS contains data about the utilisation of specialist psychiatric beds (i.e., those commissioned for use by only people with intellectual disabilities and/or autism), as well as non‐specialist beds (i.e., those that have not been commissioned exclusively for use by people with intellectual disabilities and/or autism but may nevertheless be used by this group). Further information can be found within Nisar et al. (2025).

No data were available from August 2022 to March 2023 due to a cyber incident in the NHS. Data for outcome and predictor variables were extracted from the MHSDS, and a new database was created for analysis. The data were either frequency counts or were converted to ratios representing the proportion of individuals with a particular characteristic.

Our choice of outcome variables was limited by what was available within the MHSDS but outcomes were chosen as they were indicative of psychiatric bed utilisation over time and included (a) hospital spells: total monthly number of hospital spells open at the end of the month (a spell is defined as an inpatient within a hospital at the end of the month); (b) hospital admissions: total monthly number of hospital admissions; (c) hospital discharges: total monthly number of hospital discharges; (d) community ratio: ratio of community to non‐community discharges (values > 1 indicate more community discharges relative to non‐community discharges; A non‐community discharge would include transfer to another type of ward, and a community discharge is discharge from hospital and into the community); (e) length of stay—under 2 Years: total number of patients with a length of stay under 2 years; (f) length of stay—over 2 Years: total number of patients with a length of stay over 2 years; and (g) restraints: total monthly number of distinct patients subject to restraints including physical and chemical.

Our predictor variables were a range of sociodemographic, clinical and service‐related factors thought likely to relate to psychiatric bed utilisation. Due to the number of observations available, we opted to collapse variable levels or created ratios to preserve statistical power. Our chosen predictors were (a) age—under 18: total monthly number of inpatients under the age of 18; (b) age—over 18: total monthly inpatients over the age of 18. There is evidence that children with intellectual disabilities are less likely to be admitted to psychiatric hospitals (Patil et al. 2013); (c) ethnicity ratio: ratio of White to non‐White inpatients (values > 1 indicate more White inpatients relative to non‐White discharges). There is evidence that non‐White individuals with intellectual disabilities are less likely to access psychiatric services (Durà‐Vilà and Hodes 2012); (d) ward security ratio: ratio of inpatients in forensic wards to those in acute wards (values > 1 indicate more inpatients in forensic wards relative to inpatients in acute wards). Those detained within a forensic ward may stay longer in hospital due to risk, (e) mental health ward stays: total monthly number of inpatients in mental health wards. These are non‐specialist wards that may not be experienced with providing mental health care to autistic people or those with intellectual disabilities; (f) learning disability ward stays: total monthly number of inpatients in intellectual disability wards. These are specialist wards where care is provided by professionals experienced in working with people with intellectual disabilities, (g) legal status—informal: total number of monthly informal inpatients meaning they are not detained under the Mental Health Act, 1983, and therefore may have a shorter length of stay; (h) legal status—Part II: total number of monthly inpatients detained under Part II of the Mental Health Act, 1983, who are detained due to crisis or for treatment and may have a shorter length of stay; (i) legal status—Part III total number of monthly inpatients detained under Part III of the Mental Health Act, 1983 (with and without restrictions) who may have a longer length of stay due to restrictions.

### 2.2 Statistical Analysis

Psychiatric bed utilisation over time was examined using linear regression, and the intercept, slope and *R* ^2^ were reported. Generalised least‐squares regression with either moving average (MA) or auto‐regressive (AR) errors was fitted to the data to model relationships between explanatory variables and outcomes. Model error structure was diagnosed using autocorrelation (ACF) and partial autocorrelation plots (pACF) from linear regressions which were fitted in the first instance to each outcome (see Table S1 for linear regression output). AR and MA processes differ in the following way: the AR process regresses the outcome on its own lagged values, whereas the MA process is a linear combination of previous error terms (Harrell 2016). The number of terms is a function of the lag determined by the ACF and pACF plots. An AR function will show decay towards zero in the ACF plot, and pACF will cut off after the chosen lag, whereas the MA will become zero after the chosen lag in ACF, but pACF will decay towards zero (Chatfield and Xing 2019).

## 3 Results

Descriptive statistics for our outcome and predictor variables are found in Tables 1 and 2, respectively.

**TABLE 1:** Descriptive statistics for outcome variables. Data are patient counts.

Outcome	*N* (observations)	Mean (SD)	Min	Max	Skew
Hospital spells	63	3498.97 (197.79)	3120.00	3870.00	0.06
Hospital admissions	63	1209.37 (220.71)	680.00	1695.00	0.32
Hospital discharges	63	1303.25 (214.55)	835.00	1780.00	0.21
Community discharge (ratio)	63	6.51 (4.43)	4.11	39.40	7.01
Length of stay—under 2 years	64	2232.11 (171.17)	1900.00	2775.00	0.10
Length of stay—over 2 years	64	1262.46 (130.16)	882.50	1440.00	−0.97
Restraints	55	463.09 (95.84)	330.00	695.00	0.88

**TABLE 2:** Descriptive statistics for predictor variables. Data are either patient counts or ratios.

Predictor	*N*	Mean (SD)	Min	Max	Skew
Age—under 18	63	205.32 (60.03)	150.00	575.00	3.78
Age—over 18	64	3166.41 (480.47)	1580.00	3795.00	−2.04
Ethnicity (ratio)	64	4.35 (0.33)	2.68	4.97	−1.84
Ward security (ratio)	64	0.64 (0.11)	0.53	0.93	1.39
Mental health ward stays	64	1557.11 (140.36)	1230.00	1960.00	0.86
Learning disability ward stays	64	1347.42 (270.90)	835.00	1755.00	−0.45
Legal status—informal	64	1011.25 (168.30)	755.00	1335.00	0.20
Legal status—Part II	64	1481.41 (99.87)	1275.00	1725.00	−0.07
Legal status—Part III	64	914.38 (59.98)	845.00	1125.00	1.86

### 3.1 Hospital Spells

**FIGURE 1 to 3:**
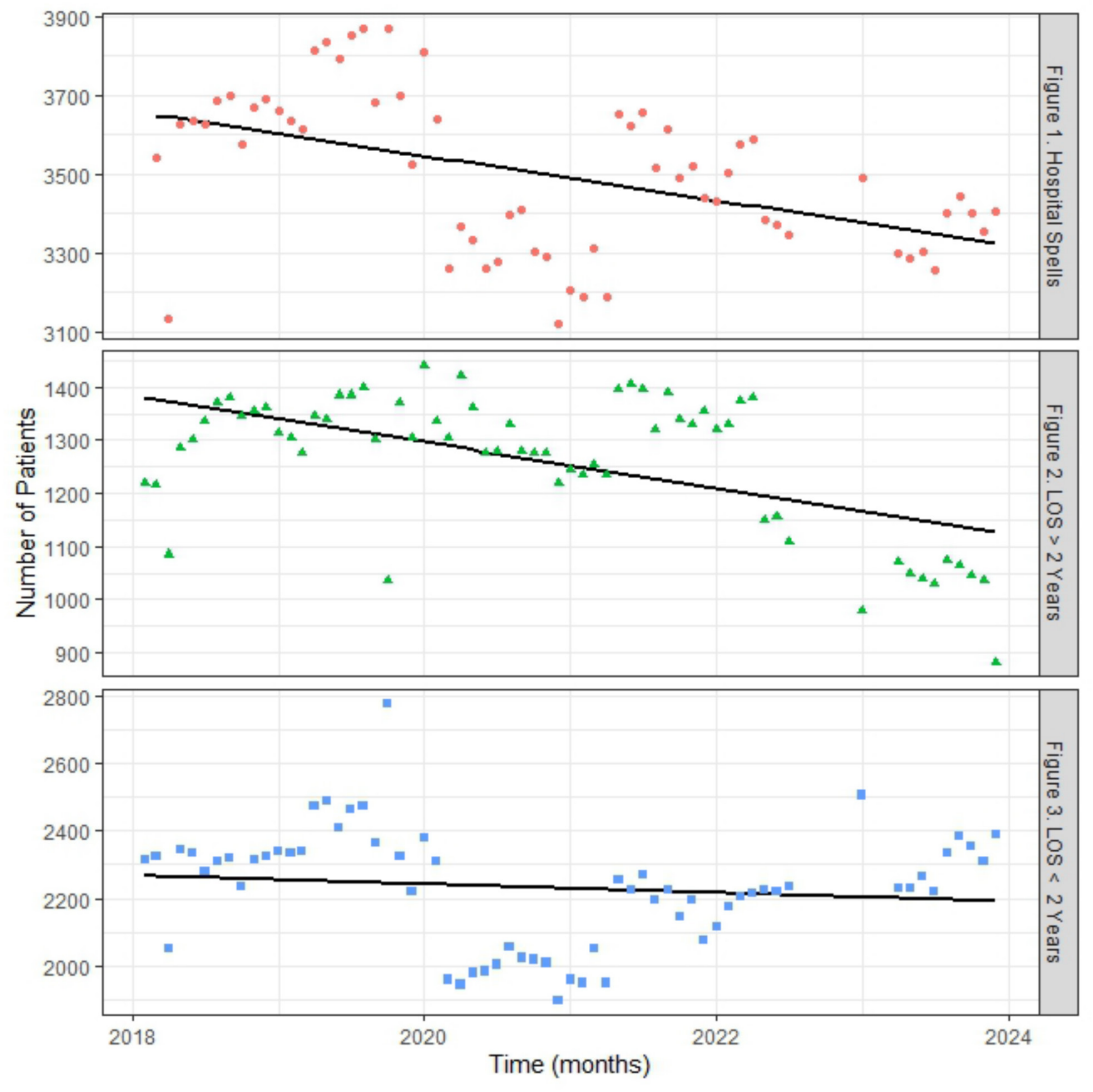
Monthly number of (a) hospital spells, (b) LOS over 2 years and (c) LOS under 2 years over time.

Considering the number of hospital spells, these reduced over time. There was an average reduction of 4.55 patients per month, with an initial monthly count in Feb 2018 (intercept) of 3654.80 (*R* ^2^ = 0.20) (Figure 1). We also examined whether the number of hospital spells varied according to length of stay. For those with a longer length of stay, and specifically, for inpatients with a length of stay over 2 years, there was a reduction of an average of 3.65 patients per month with an initial monthly count in Feb 2018 (intercept) of 1385.70 (*R* ^2^ = 0.32) (Figure 2). However, the number of inpatients with a length of stay under 2 years remained stable over time (Figure 3), with a slope value of −0.91 and an initial monthly count in Feb 2018 (intercept) of 2263.71 (R2 = −0.00) (Figure 2). These findings indicated that the reduction in hospital spells over time was due to a reduction in the number of inpatients with length of stay longer than 2 years.

While overall there was a reduction in the number of hospital spells, periods of time where there was an increase in the number of hospital spells were significantly associated with an increase in the number of inpatients aged under 18 years, more inpatients within forensic wards, relative to non‐forensic wards, increased mental health ward stays and intellectual disability ward stays, as well as an increase in the number of informal inpatients, and those detained under Part II and Part III of the Mental Health Act, 1983, Table 3. Periods where there had been an increase in the number of hospital spells were also associated with a decrease in the number of White inpatients relative to non‐White inpatients.

**TABLE 3:** AR models of the relationship between various predictor variables and outcome variables within the Mental Health Services Dataset.

	Hospital spells	Hospital admissions	Hospital discharges	Community ratio	Length of stay—under 2 years	Length of stay—over 2 years	Restraints (count of people)
	Estimate [*95% CI*]	Estimate [*95% CI*]	Estimate [*95% CI*]	Estimate [*95% CI*]	Estimate [*95% CI*]	Estimate [*95% CI*]	Estimate [*95% CI*]
(Intercept)	44.383	89.517	1692.551 *	21.888	−233.253	243.192	−72.115
	[−39.909, 128.675]	[−1220.4921399.526]	[431.1382953.965]	[−18.361, 62.136]	[−819.517, 353.012]	[−398.366, 884.750]	[−701.710, 557.480]
Age—under 18	0.252 ***	0.359	0.409	−0.003	0.131	−0.091	0.230
	[0.214, 0.289]	[−0.226,0.945]	[−0.093,0.910]	[−0.023, 0.017]	[−0.132, 0.393]	[−0.379, 0.198]	[−0.067, 0.526]
Age—over 18	0.006	−0.142	−0.153	0.001	−0.087 *	0.098 *	−0.178
	[−0.005, 0.016]	[−0.303,0.019]	[−0.308,0.001]	[−0.004, 0.005]	[−0.159, −0.015]	[0.020, 0.177]	[−0.405,0.049]
Ethnicity (ratio)	−13.726 *	−195.822 *	−211.482 *	−1.818	−117.342 **	110.446 **	35.003
	[−25.662, −1.790]	[−377.214,−14.430]	[−401.606,−21.357]	[−7.058, 3.422]	[−198.440, −36.244]	[21.921, 198.971]	[−32.468, 102.474]
Ward security (ratio)	131.370 ***	−608.782	−485.387	6.176	−1.261	177.069	221.771
	[69.144, 193.596]	[−1578.094360.530]	[−1645.854675.079]	[−21.458, 33.810]	[−434.439, 431.916]	[−295.261, 649.400]	[−199.031, 642.574]
Mental health ward stays	0.059 **	0.272	0.236	−0.000	0.473 **	−0.500 **	0.013
	[0.016, 0.102]	[−0.381,0.925]	[−0.415,0.887]	[−0.020, 0.020]	[0.181, 0.765]	[−0.820, −0.181]	[−0.267, 0.293]
Learning disability ward stays	0.058 **	0.948 **	−0.012	−0.022 *	0.127	−0.160	−0.094
	[0.020, 0.095]	[0.360,1.535]	[−0.738,0.715]	[−0.039, −0.005]	[−0.135, 0.390]	[−0.447,0.127]	[−0.337, 0.150]
Legal status—informal	0.896 ***	−0.068	0.220	0.018 *	0.629 ***	0.369 *	−0.149
	[0.859, 0.933]	[−0.634,0.497]	[−0.377,0.816]	[0.001, 0.034]	[0.376, 0.882]	[0.093, 0.646]	[−0.382, 0.083]
Legal status—Part 2	0.950 ***	1.252 **	0.426	−0.024	1.013 ***	−0.065	0.517 *
	[0.896, 1.004]	[0.408,2.097]	[−0.393,1.245]	[−0.050, 0.001]	[0.636, 1.391]	[−0.478, 0.348]	[0.140, 0.894]
Legal status—Part 3	0.962 ***	−0.811	0.055	0.038	0.199	0.928 *	0.311
	[0.864, 1.060]	[−2.376,0.754]	[−1.310,1.419]	[−0.008, 0.085]	[−0.501, 0.899]	[0.164, 1.693]	[−0.475, 1.097]
*N*	63	63	63	62	63	63	55
AIC	−234.870	787.814	778.150	−196.337	−339.290	−344.063	−282.541

*** *p* < 0.001;

** *p* < 0.01;

* *p* < 0.05.

### 3.2 Hospital Admissions

**FIGURE 4 to 6:**
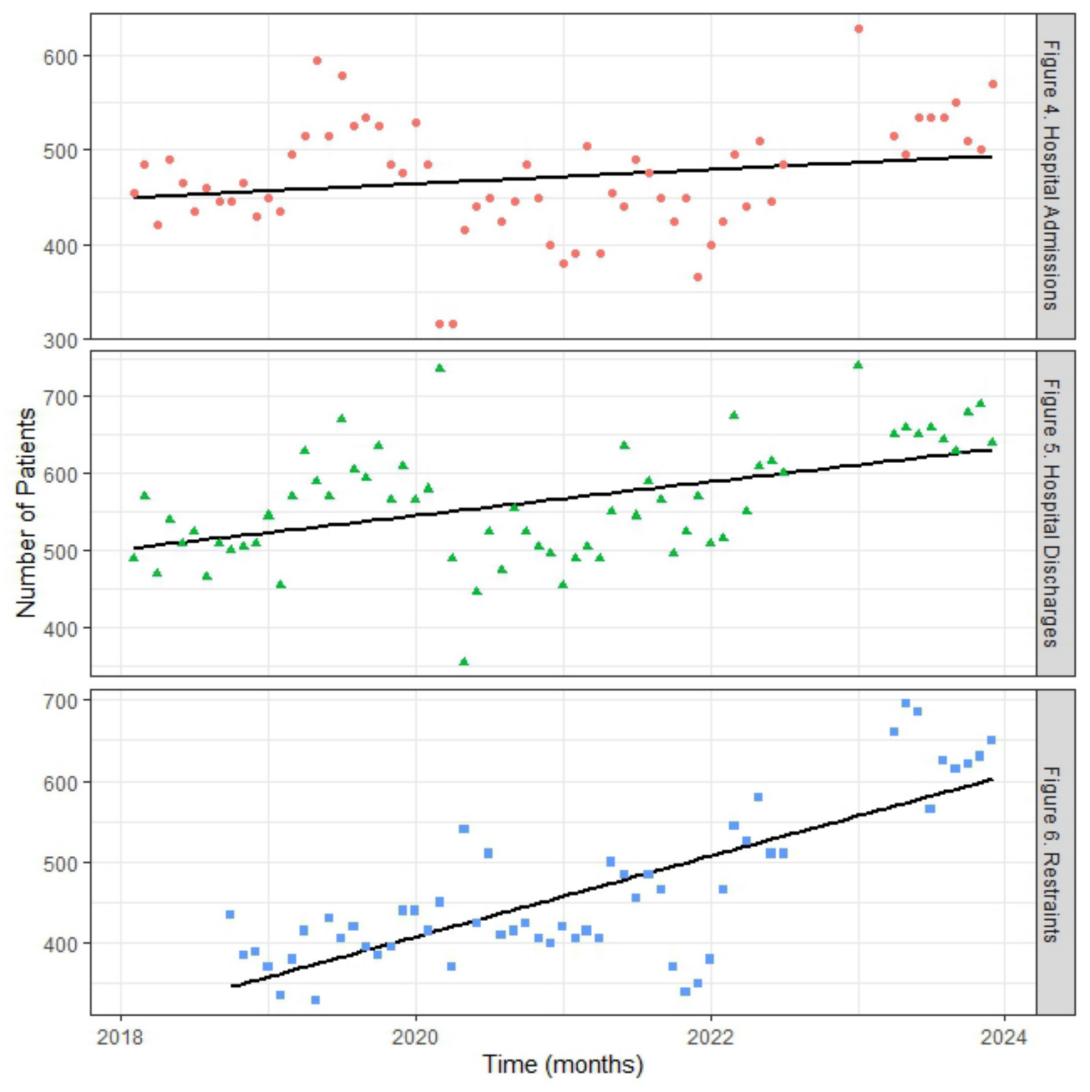
Monthly number of (a) hospital admissions, (b) hospital discharges and (c) patients restrained over time.

The number of hospital admissions decreased over time (Figure 4), with an average decrease of 5.45 patients per month and an initial monthly count in Feb 2018 (intercept) of 1396.17 (*R* ^2^ = 0.0.24). While the number of hospital admissions decreased over time, periods of time where the number of hospital admissions was higher were associated with more inpatients detained under Part II of the Mental Health Act, 1983, more intellectual disability ward stays, and a decrease in White inpatients relative to non‐White inpatients, Table 3.

### 3.3 Hospital Discharges

The number of hospital discharges also decreased over time (Figure 5), with an average decrease of 4.30 patients per month and an initial monthly count in Feb 2018 (intercept) of 1450.60 (*R* ^2^ = 0.15). Periods of time where the number of hospital discharges was higher were significantly associated with a decrease in the number of White inpatients, relative to the number of non‐White inpatients, Table 3, suggesting that White patients were being discharged more infrequently and non‐White patients were being discharged more frequently during these periods.

### 3.4 Restraints

The number of inpatients restrained increased over time (Figure 6), with an average increase of 4.14 patients per month and an initial monthly count in Feb 2018 (intercept) of 308.72 (*R* ^2^ = 0.60). Periods of time where restraints were higher were associated with an increase in the number of inpatients detained under Part II of the Mental Health Act, Table 3. No other predictors were statistically significant in this model.

### 3.5 Community Discharge Ratio

Periods of time where there had been an increase in the number of intellectual disability ward stays were associated with fewer community discharges relative to non‐community discharges. Additionally, periods where community discharges were higher were associated with increases in the number of informal inpatients who are not detained under the Mental Health Act.

### 3.6 Length of Stay (Under 2 Years)

Over time, those periods where there had been an increase in the number of inpatients with a length of stay under 2 years were significantly associated with a decrease in the number of patients that were older than 18 years, indicating older patients are less likely to have shorter stays. Periods where there had been an increase in the number of inpatients with a length of stay under 2 years were also associated with a decrease in White patients relative to non‐White patients, indicating White patients were also less likely to have shorter stays. Finally, periods with more inpatients with a length of stay of under 2 years were significantly associated with an increase in the number of mental health ward stays, the number of informal patients, and those detained under Part II of the Mental Health Act.

### 3.7 Length of Stay (Over 2 Years)

Conversely to the length of stay under 2 years, periods of time where there were more inpatients within the hospital who had stayed for longer than 2 years were associated with an increase in the number of individuals that were older than 18 years, Table 3. This was also associated with an increase in the number of White inpatients relative to non‐White inpatients indicating more White patients stayed in the hospital for longer than 2 years relative to non‐White patients. Periods where the number of inpatients with a length of stay over 2 years were higher were also associated with an increase in the numbers of informal patients and detention under Part III of the Mental Health Act, respectively, as well as a decrease in the number of mental health ward stays.

## 4 Discussion

The aim of this study was to examine psychiatric bed utilisation over time and to explore how a series of chosen sociodemographic, clinical and service‐related predictor variables from MHSDS influenced admissions and discharges of autistic people and people with intellectual disabilities to any type of psychiatric bed across England, using time series modelling. Our results indicated that the number of hospital spells (i.e., the number of inpatients) decreased from February 2018 to January 2024 by an average of 4.55 inpatients per month. Hospital discharges and the number of inpatients staying for longer than 2 years were also found to have decreased over time, while the number of inpatients subject to physical restraints increased. However, the number of stays under 2 years remained stable.

There were several associations of note between predictor and outcome variables. Firstly, the increase in the number of hospital spells was associated with increases in the numbers of inpatients under the age of 18, suggesting there is a rising number of autistic young people and young people with intellectual disabilities utilising psychiatric beds over time. It is important to understand what might be driving this increase, as while this could be attributable to increased prevalence of mental ill health, it is more likely to reflect a lack of appropriate community services that prevent both crisis and admission, as outlined in the ‘Psychiatric services for young people with intellectual disabilities’ report by the Royal College of Psychiatrists (2016). Melvin et al. (2022) noted that there are few published studies about models of inpatient care and associated outcomes including studies about inpatient safety for children and young people with intellectual disabilities and/or autism relative to the literature about inpatient services for adults.

We also found an association with ethnicity and the number of hospital spells, as periods of time where hospital stays and admissions were higher were associated with more non‐White inpatients relative to White inpatients overall. However, periods of time when there were more White inpatients, relative to non‐White inpatients, were associated with longer lengths of stay and fewer discharges. These findings suggest that White individuals stayed longer in hospital and were less likely to be discharged. However, periods when the number of hospital stays and admissions were higher were associated with an increased proportion of non‐White inpatients, who stayed for shorter periods. There is evidence that South Asian children, adolescents and adults with intellectual disabilities are less likely to access community mental health services relative to their White counterparts in the United Kingdom, while internationally, those from non‐White backgrounds may be less likely to access psychiatric services (Durà‐Vilà and Hodes 2012). Cultural barriers that reduce access to community psychiatric services for autistic people and people with intellectual disabilities may prevent early intervention and future admission avoidance leading to an increased probability of experiencing crisis, which may explain the relationship between increased admissions for shorter periods for non‐White individuals.

For inpatients detained under Part II of the Mental Health Act, 1983, there were several associations with outcome variables—periods where there were increased numbers of inpatients detained under Part II were positively associated with more hospital admissions, more discharges to the community, and more stays under 2 years. Periods where there was an increase in those detained under Part II may reflect inadequate or inappropriate community services, precipitating a crisis for some, leading to detention in hospital. It was concerning that there was also an association between increases in the number of patients detained under Part II and increases in number of inpatients subject to restraints. This may be due to an increase in the number of patients experiencing acute crisis, who subsequently are detained under Part II, which consequently prompts greater use of physical restraints and medication. Over time, it may be the case that those admitted have increased acuity due to attempts to avoid hospital admission. Increased acuity has the potential to lead to greater use of physical restraint and medication.

Further, we also found that increased hospital stays were associated with an increase in the number of informal patients and patients detained under Part III. It has been suggested that while there has been an increase in the number of people accessing mental health services over time within the United Kingdom, austerity and the economic recession experienced from 2008 onwards may explain the increase in the number of informal admissions and detentions under Part II of the Mental Health Act, 1983 (Smith et al. 2020) as autistic individuals and those with intellectual disabilities and their families would have felt these effects. Detention under Part III of the Mental Health Act was associated with a length of stay longer than 2 years, which is unsurprising as detention under Part III can only be ordered by a Crown Court judge and serves as an alternative to imprisonment. Therefore, those placed under a restriction order who can only be discharged from hospital by a Mental Health Tribunal or those presenting with a continuing risk of committing a criminal offence, discharge may take longer. It is worth noting that there is some evidence that closing NHS beds for people with intellectual disabilities may lead to an increase in the number of prisoners in England in the future (Wild et al. 2022).

### 4.1 Clinical Implications

The findings of this time series analysis indicated that various factors, including demographic factors such as age and ethnicity, as well as inpatient's legal status, can influence psychiatric bed utilisation by autistic people and people with intellectual disabilities. Therefore, decision‐making regarding the closure of these psychiatric beds should be informed by the distinct needs of the population. Admissions for patients detained under Part II of the Mental Health Act increased, which indicates there is a demand for these beds, which may be driven by a policy of austerity; if reductions are made in the number of psychiatric beds available while the need for them remains, this may have a detrimental impact if adequate alternative provisions are not available in the community. For example, if neither psychiatric beds nor suitable community services are available, this may result in individuals experiencing repeated crises, and for some, detention under Part III of the Mental Health Act, or being sent to prison, may occur if they become involved in criminal proceedings. The goal to reduce psychiatric bed utilisation must be weighed against what is most appropriate and beneficial for autistic people and people with intellectual disabilities who have mental illness.

### 4.2 Strengths, Limitations and Future Research

A strength of this study is we made use of MHSDS to explore what factors can influence psychiatric bed utilisation by autistic people and people with intellectual disabilities. This is a relatively large dataset of English national data allowing for the exploration of relationships between psychiatric bed utilisation and a series of sociodemographic, clinical, and service‐related variables which help inform mental health service development. There are also some weaknesses of the study to consider. Due to the relatively small number of observations, a limited number of predictor variables from the Mental Health Services Data Set could be included in the auto‐regressive model, meaning associations with potentially relevant factors (i.e., Care [Education] and Treatment Reviews) could not be examined in the current analysis. Also, we were unable to develop separate statistical models for inpatients with intellectual disabilities and autistic inpatients without intellectual disabilities. Additionally, as this time series study utilises observational data, no inferences can be made regarding causation. This is a problem with the utilisation of aggregated patient data; individual level patient data over time would allow for alternative statistical models that are more powerful.

Future research should seek to explore how psychiatric beds can be used more effectively and efficiently, in conjunction with community‐based services with consideration given to the specific needs of different demographics (e.g., young people and ethnic minorities) and how these factors interact with bed utilisation. It would also be beneficial to use individual patient level data to explore the trajectories of inpatients of autistic people and people with intellectual disabilities who have accessed psychiatric beds over time, as this would provide valuable information regarding service utilisation and long‐term outcomes for this population. Finally, it would be helpful in the future to consider generating separate statistical models for inpatients with intellectual disabilities and autistic inpatients without intellectual disabilities.

## Ethics Statement

Ethical approval was obtained through the University of Warwick Humanities & Social Sciences Research Ethics Committee (HSSREC 124/23‐24).

## Conflicts of Interest

The authors declare no conflicts of interest.

## Supporting information


**Table S1** Linear models of the relationship between various predictor variables and outcome variables within the Mental Health Services Dataset

## Data Availability

The data that support the findings of this study are available on reasonable request from the corresponding author.
