# Supplementary material for: Factors Affecting Psychiatric Bed Utilisation by People With Intellectual Disabilities: A Time Series Analysis Using the English National Mental Health Services Data Set
Source: J Intellect Disabil Res. 2025 Jun 23;69(11):1251–60. doi: 10.1111/jir.70003 (PMC12576377; doi:10.1111/jir.70003)
Supplement: Supplementary file 1 — Table S1 Linear models of the relationship between various predictor variables and outcome variables within the Mental Health Services Dataset [file JIR-69-1251-s001.docx]

| Table S1. Linear models of the relationship between various predictor variables and outcome variables within the Mental Health Services Dataset | | | | | | | |
| --- | --- | --- | --- | --- | --- | --- | --- |
|  | | | | | | | |
|  | Hospital Spells | Hospital Admissions | Hospital Discharges | Community Discharges | Length of Stay – Under 2 Years | Length of Stay – Over 2 Years | Restraints (Count of People) |
|  | Estimate [*95% CI*] | Estimate [*95% CI*] | Estimate [*95% CI*] | Estimate [*95% CI*] | Estimate [*95% CI*] | Estimate [*95% CI*] | Estimate [*95% CI*] |
| (Intercept) | 20.18 | -22.12 | 90.83 | 20.44 | -420.11 | 473.48 | -318.87 |
|  | [-60.87,101.24] | [-414.76,370.51] | [-455.76,637.42] | [-19.63,60.51] | [-1046.02,205.80] | [-201.33,1148.28] | [-943.82,306.07] |
| Age - Under 18 | 0.24 *** | 0.15 | 0.19 | -0.00 | 0.14 | -0.12 | 0.18 |
|  | [0.20,0.28] | [-0.05,0.35] | [-0.09,0.47] | [-0.02,0.02] | [-0.18,0.46] | [-0.47,0.23] | [-0.14,0.51] |
| Age - Over 18 | 0.01 | -0.05 * | -0.02 | 0.00 | -0.11 ** | 0.12 ** | -0.17 |
|  | [-0.00,0.01] | [-0.10,-0.01] | [-0.09,0.04] | [-0.00,0.01] | [-0.18,-0.03] | [0.04,0.19] | [-0.40,0.07] |
| Ethnicity (Ratio) | -12.01 * | -40.33 | -13.15 | -1.80 | -98.44 * | 85.62 | 44.11 |
|  | [-22.44,-1.57] | [-90.88,10.22] | [-83.52,57.23] | [-6.91,3.31] | [-179.03,-17.85] | [-1.26,172.50] | [-20.81,109.02] |
| Ward Security (Ratio) | 162.54 *** | -247.89 | -42.87 | 4.89 | 6.82 | 184.24 | 91.81 |
|  | [107.34,217.75] | [-515.32,19.55] | [-415.17,329.43] | [-22.23,32.01] | [-419.50,433.15] | [-275.38,643.87] | [-323.88,507.49] |
| Mental Health Ward Stays | 0.07 ** | 0.22 * | 0.08 | 0.00 | 0.55 *** | -0.59 *** | 0.11 |
|  | [0.03,0.11] | [0.02,0.41] | [-0.18,0.35] | [-0.02,0.02] | [0.24,0.86] | [-0.92,-0.26] | [-0.17,0.38] |
| Learning Disability Ward Stays | 0.06 *** | 0.17 * | -0.12 | -0.02 * | 0.24 | -0.28 | -0.08 |
|  | [0.03,0.10] | [0.01,0.34] | [-0.35,0.10] | [-0.04,-0.00] | [-0.02,0.50] | [-0.56,0.00] | [-0.32,0.16] |
| Legal Status - Informal | 0.90 *** | 0.02 | 0.16 | 0.02 * | 0.59 *** | 0.43 ** | -0.19 |
|  | [0.86,0.93] | [-0.14,0.18] | [-0.06,0.39] | [0.00,0.03] | [0.33,0.84] | [0.15,0.70] | [-0.42,0.04] |
| Legal Status - Part 2 | 0.97 *** | 0.39 ** | 0.29 | -0.02 | 1.13 *** | -0.19 | 0.37 |
|  | [0.92,1.02] | [0.15,0.64] | [-0.05,0.63] | [-0.05,0.00] | [0.74,1.52] | [-0.61,0.24] | [-0.02,0.76] |
| Legal Status - Part 3 | 0.91 *** | -0.22 | 0.03 | 0.04 | -0.07 | 1.19 ** | 0.71 |
|  | [0.82,1.00] | [-0.68,0.23] | [-0.60,0.66] | [-0.01,0.08] | [-0.80,0.65] | [0.41,1.97] | [-0.11,1.52] |
| N = | 63 | 63 | 63 | 62 | 63 | 63 | 55 |
| R^2^ = | 1.00 | 0.59 | 0.50 | 0.20 | 0.87 | 0.74 | 0.76 |
| AIC = | 461.62 | 660.41 | 702.10 | 365.78 | 719.17 | 728.65 | 601.28 |
| *** p < 0.001; ** p < 0.01; * p < 0.05. | | | | | | | |
